# Supplementary figures and images for: Reference genome for the Northern bat (Eptesicus nilssonii), a most northern bat species
Source: J Hered. 2023 Oct 4;115(1):149–54. doi: 10.1093/jhered/esad056 (PMC10838120; doi:10.1093/jhered/esad056)

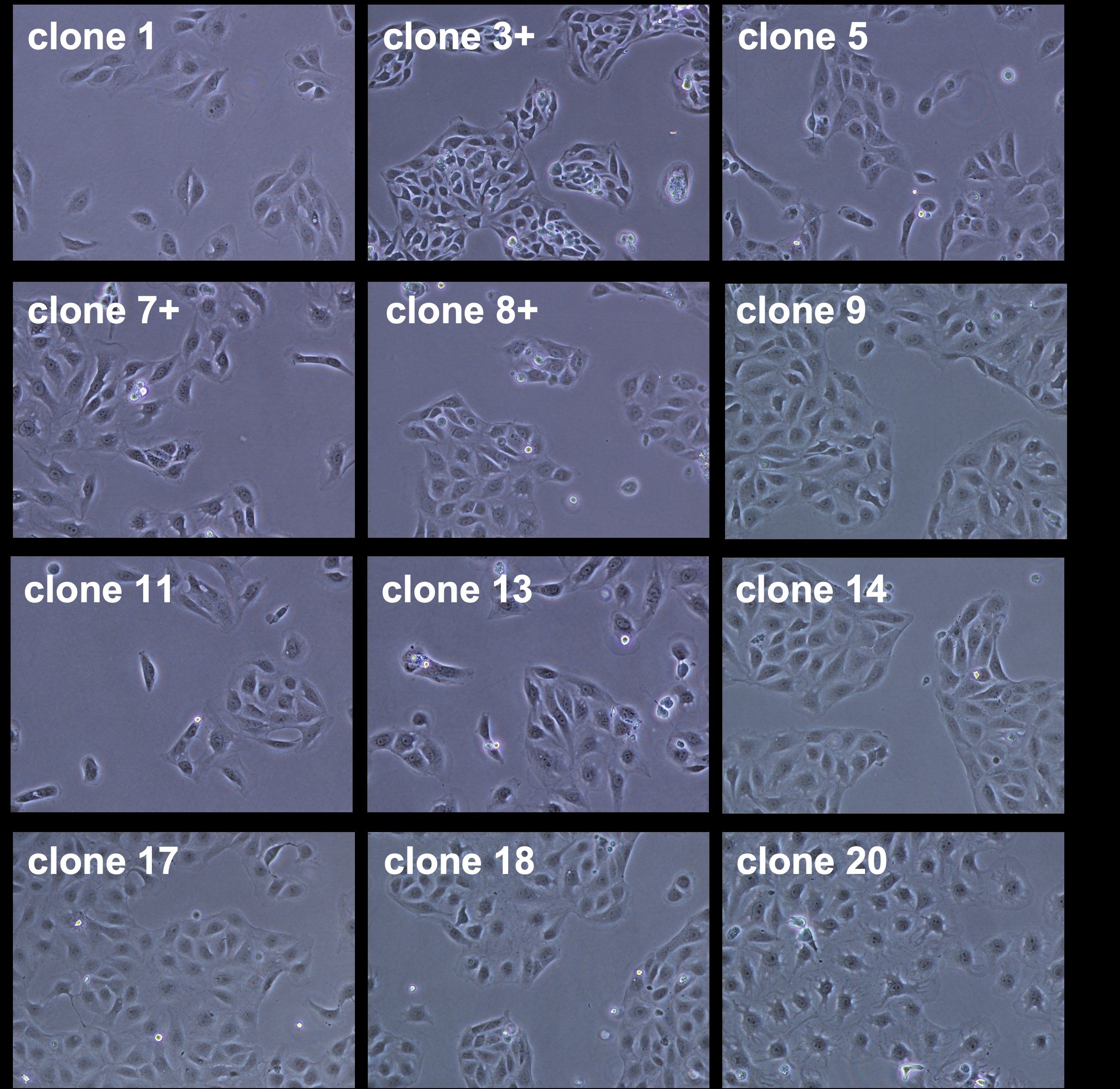

Supplement: esad056_suppl_Supplementary_Figure_S1 [file esad056_suppl_supplementary_figure_s1.jpeg]
